# Effect of Ultrasonic Surface Rolling Process on the Hot Compression Behavior of Inconel 718 Superalloy at 700 °C

**DOI:** 10.3390/nano9040658

**Published:** 2019-04-25

**Authors:** Zhiyan Sun, Shuai Ren, Timin Hu, Bo Li

**Affiliations:** 1 School of Materials Science and Engineering, Tsinghua University, Beijing 100084, China; szy14@mails.tsinghua.edu.cn (Z.S.); hutm14@mails.tsinghua.edu.cn (T.H.); 2 Advanced Materials Institute, Graduate School at Shenzhen, Tsinghua University, Shenzhen 518055, China; 3 HBIS Group Technology Research Institute, Shijiazhuang 050000, China

**Keywords:** inconel 718 superalloy, ultrasonic surface rolling process, hot compression

## Abstract

A series of hot compression tests at the temperature of 700 °C were applied to study the effect of the ultrasonic surface rolling process (USRP) on the hot compression behavior of Inconel 718 superalloy. The results indicated that the USRP-treated samples exhibited a better ability to withstand axial hot compression than the untreated samples. After the hot compression process, the size of the matrix grains was slightly decreased, and the volume fraction of ultra-fine recrystallized grains in the near-surface regions was increased for the USRP-treated samples. In addition, for USRP-treated samples, a large number of γ″ phases with size less than 100 nm were precipitated within the broadened grain boundaries in the near-surface regions rather than the inner grains. The enhanced ability to withstand axial compression at 700 °C for USRP-treated samples was related to the ultra-fine microstructure induced by USRP, combined with the precipitation of nano-γ″ phases within broadened grain boundaries and the increase of ultra-fine recrystallized grains in the near-surface regions during the hot compression process.

## 1. Introduction

Owing to the excellent corrosion resistance and high strength at elevated temperatures, nickel-based superalloy Inconel 718 is widely applied to aero engines, industrial gas turbines, etc. [1,2,3]. The metastable body-centered tetragonal γ″ phase and spherical γ′ phase are the main strengthening phases of the Inconel 718 superalloy [4,5,6]. As a precipitation-strengthened superalloy, Inconel 718 is the most popular commercial superalloy in the manufacturing of key parts for aero engines [7,8]. With the development of society, more critical requirements for the high temperature performance of hot components were put forward by the designers of aero engines [9], such as high strength and good fatigue resistance at high temperature. However, it is difficult to meet these requirements using traditional treatment methods [10].

The ultrasonic surface rolling process (USRP) is an advanced surface strengthening technology that impacts the specimen’s surface with ultrasonic energy through a scrollable rolling tip [11]. Based on severe surface plastic deformation induced by USRP, a strengthened layer in near-surface regions with nano-sized grains, high surface hardness, and compressive residual stress can be obtained after USRP [9]. Thus, significant improvements in the surface strength, wear resistance, and fatigue life could be achieved for Inconel 718. In addition, USRP has been used in several other materials [12,13,14,15,16,17]. Amanov et al. [18] reported that an improved fatigue life of Inconel 718 superalloy could be obtained after USRP treatment. The enhancement in fatigue life was closely related to the compressive residual stresses introduced in the near-surface regions. In addition, it has been observed that these enhancements in the near-surface regions have certain stability at elevated temperatures [19,20,21]. However, in most studies, the near-surface regions after USRP treatment were in a radial compressive stress state. In the service process, the hot-section component pieces work under complex stresses such as centrifugal force, alternating stress, thermal stress, etc. Therefore, a study of the ability of USRP-treated Inconel 718 to withstand high-temperature deformation is of crucial importance. However, little research has been done on the axial compression of the near-surface regions, especially at elevated temperatures.

Thermal simulation is an effective method to simulate the axial force of the specimen under elevated temperature conditions. Boehlert et al. [22] revealed that cold-reduction could give rise to creep strength by subjecting the Inconel 718 superalloy to cold-rolling. Zhang et al. [23] proposed that the cold rolling process could have significant effects on the hot tensile behavior of Inconel 718 superalloy at 650 °C. Zhang et al. [5] reported that the precipitation behaviors of γ″ and δ phases were tailored by hot deformation temperatures. The precipitation of γ″ and δ phases was accelerated at the hot deformation temperatures of 800 and 900 °C, respectively. The hot deformation behaviors of a nickel-based alloy with various contents of the δ phase were investigated by Lin et al. [24]. The results revealed that the microstructure evolution and hot compression behaviors were related to the content of the δ phase as well as the thermomechanical parameters. Tan et al. [25] investigated the hot compression behaviors of Inconel 718 with fine grains by hot compression tests with the true strain rate range of 0.1–10 s^−1^ and the temperature range of 950–1150 °C. The hot deformation behaviors are closely related to initial grain size, the morphologies of the secondary phases, and hot deformation parameters (deformation temperature, deformation degree, and strain rate) [26,27,28,29,30]. Therefore, the ultra-fine grains in the near-surface regions obtained by USRP would undoubtedly affect the ability of Inconel 718 to withstand axial hot compression. Therefore, it is necessary to investigate the effects of USRP on the hot compression behavior of Inconel 718 superalloy.

In this study, a series of hot compression experiments were applied to study the effect of USRP on the hot compression behavior of Inconel 718 superalloy at 700 °C. The variations of true stress–strain curves obtained by hot compression experiments were characterized, and the microstructure before and after deformation was analyzed. According to the experimental results, we discuss the superalloy’s ability to withstand axial hot compression as well as the evolution of ultra-fine microstructure obtained by USRP during the hot compression process at 700 °C.

## 2. Experimental Procedures

The as-received Inconel 718 superalloy was in a fully annealed state with the chemical composition of 52.5Ni–18.84Cr–18.33Fe–5.06Nb–3.08Mo–1Ti–0.05Al. Before the USRP, the cylindrical Inconel 718 samples were prepared by turning and grinding the specimens to a length of 120 mm and a diameter of 10 mm to ensure consistent surface morphologies as much as possible. Subsequently, USRP was conducted on a self-designed platform which was modified according to the computer numerical control (CNC) lathe. Table 1 shows the processing parameters of USRP in this study.

**Figure 1:**
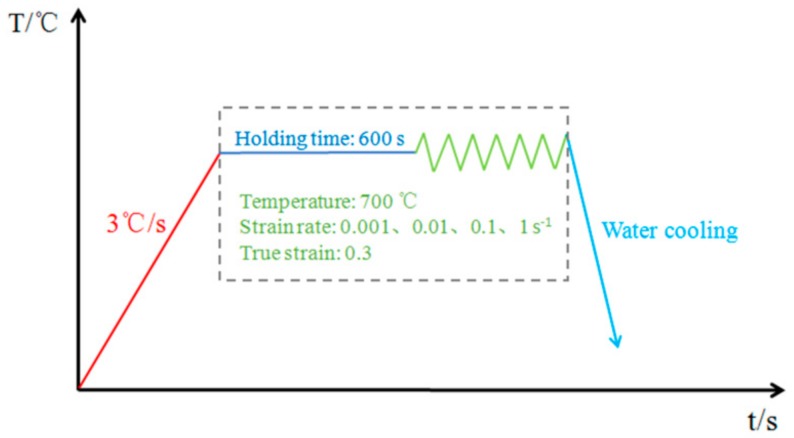
Schematic diagram of the hot compression test in this work.

After USRP, hot compression samples with a length of 15 mm were cut along the length direction from the rods by a traditional lathe. Hot compression tests were carried out on the Gleeble 3800 thermomechanical simulator at a temperature of 700 °C and true strain rates of 0.001, 0.01, 0.1, and 1 s^−1^. A NiCr–NiSi thermocouple spot was utilized to measure and control the temperature. A graphite lubricant was coated on the surfaces of specimens. Tantalum foil was used to help reduce friction and avoid adhesion. Hot compression tests were carried out under vacuum. As shown in Figure 1, the samples were heated to 700 °C at 3 °C/s and soaked for 10 min to remove the thermal gradient before hot compression tests. The stress–strain data were automatically recorded by the testing system during deformation. When the true strain reached 0.3, the specimens were immediately quenched in water to hold the microstructure. All the compressed samples were cut parallel to the compression axis by a wire-electric discharge machine to observe the microstructure evolution. A Zeiss Ultra 55 scanning electron microscope (SEM) with electron back-scattered diffraction (EBSD) equipment and a Zeiss Lsm700 laser scanning confocal microscope (Carl Zeiss, Oberkochen, Germany) were used to characterize the microstructure evolution. In order to characterize optical and SEM morphologies, the grinded samples were mechanically polished for 10 min using diamond polish, and then chemically etched (HCl:HNO_3_:HF:H_2_O = 50:10:2:38) for 1–2 min at room temperature. In addition, the samples used for EBSD characterization were prepared by an Ion grinder (Hitachi IM4000, Hitachi, Tokyo, Japan) after being mechanically polished for 10 min.

## 3. Results and Discussion

### 3.1. Hot Compression

**Figure 2:**
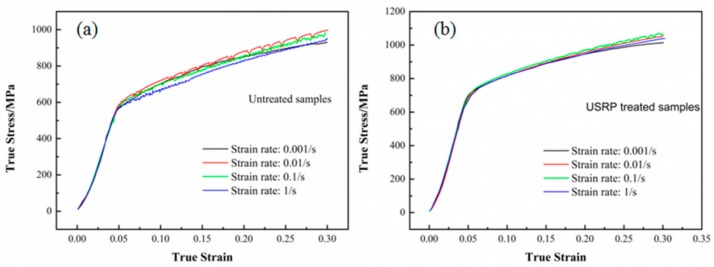
True stress–strain curves of the hot compressed samples under different strain rates at 700 °C: (**a**) Untreated samples, (**b**) Ultrasonic surface rolling process (USRP)-treated samples.

True stress–strain curves of different true strain rates were obtained to characterize the samples’ ability to withstand axial pressure. Figure 2 shows the true stress–strain curves of the hot compressed untreated and USRP-treated samples at 700 °C. As can be seen from Figure 2, the hot compression true stress–strain curves of the untreated (Figure 2a) and USRP-treated samples (Figure 2b) at different strain rates were all typical uniform elastic plastic curves, and exhibited similar behavior. Two deformation stages were observed during the hot compression process. The true stress–strain curves in the beginning stage were straight lines, which indicated uniform elastic deformation. As the compressive stress reached the yield strength, the compression curves were parabolic, which indicated uniform plastic deformation.

During hot compression, samples undergo work hardening, dynamic recovery, dynamic recrystallization, and precipitation strengthening [27,31]. As can be assumed, the initial rapid rise in true stress was related to the increase of dislocation density and the formation of poorly developed subgrain boundaries, as a result of work hardening and dynamic recovery [32]. In the case of the Inconel 718 superalloy, the work softening caused by dynamic recovery was small due to its relatively low stacking fault energy. The small/sharp drop “after yield peak” observed in some papers [25,33,34,35] did not appear in this work, since the hot compression temperature of 700 °C was far below the temperature range of 900–1100 °C used in those studies. There was no ultimate strength in any of the stress–strain curves. The stress kept increasing until the strain reached the maximum of 0.3. The peak stresses of the stress–strain curves are listed in Table 2. When the true strain rates were 0.01 and 0.1 s^−1^, the peak stress values were higher than the true strain rates of 0.001 and 1 s^−1^ for both untreated samples and USRP-treated samples. This indicated that the untreated and USRP-treated Inconel 718 superalloy could obtain an optimal ability to resist axial compression at 700 °C. This is similar to the conclusions that certain temperatures and strain rates are the optimum processing parameters for Inconel 718 superalloy reported by some references [25,29,36].

**Figure 3:**
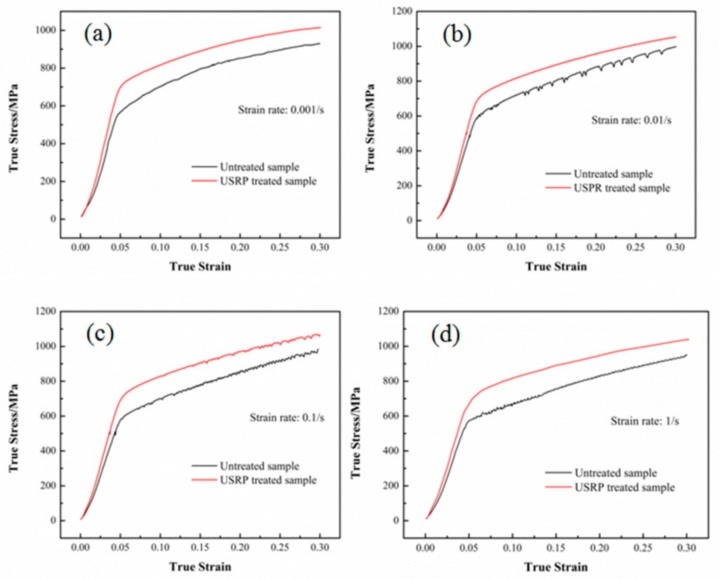
True stress–strain curves of the hot-compressed USRP-treated samples and untreated samples at different true strain rates: (**a**) 0.001 s^−1^, (**b**) 0.01 s^−1^, (**c**) 0. 1 s^−1^, (**d**) 1 s^−1^.

In addition, compared with the untreated samples, the peak stress values (Table 2) of the USRP-treated samples were increased by 9.3%, 5.6%, 8.3%, and 9.1% at the true strain rates of 0.001, 0.01, 0.1, and 1 s^−1^, respectively. To further study the ability of USRP-treated samples and untreated samples to withstand axial compression at 700 °C, the true stress–strain curves for these samples at the same true strain rate are depicted in Figure 3. As can be seen from Figure 3, the true stress values of the USRP-treated samples were overall higher than those of the untreated samples during the hot compression process. The work hardening process was obvious for both untreated and USRP-treated samples. The relationship between the true strain and true stress in the uniform elastic deformation stage can be expressed as:

(1) $$\sigma_{e}=E\varepsilon_{e},$$

Where $\sigma_{e}$, *E*, and $\varepsilon_{e}$ are the elastic stress, Young’s modulus, and elastic strain, respectively. The Young’s modulus *E* represents the ability to resist elastic distortion. In this study, the Young’s moduli E for the untreated and USRP-treated samples were calculated to be 115 and 140 GPa, respectively. Due to the absence of prior USRP treatment, it could be assumed that the near-surface regions had the same dislocation density as the core region. The structure of untreated samples was represented by the lower E value of 115 GPa. Once prior USRP-treatment was carried out, the dislocation density and special grain boundaries in the near-surface regions were increased [37], which could improve the ability to significantly hinder grain boundary movement and dislocation slipping in near-surface regions during hot compression, resulting in the boosted E value of 140 GPa. Similar results indicating that prior cold deformation could improve the Young’s modulus of Inconel 718 superalloy were reported by Zhang et al. [23].

The relationship between the true strain and true stress in the uniform plastic deformation stage can be expressed as:

(2) $$\sigma_{p}=K{\varepsilon_{p}}^{n},$$

where $\sigma_{p}$, *K*, $\varepsilon_{p}$, and n are the plastic stress, strength factor, plastic strain, and strain hardening exponent, respectively. The strain hardening exponent n, which determines the maximum uniform plastic strain before the occurrence of fracture, reflects the straining hardening behavior during uniform plastic deformation. In this study, the difference in true stress value between the untreated and USRP-treated samples decreased as the true strain value increased during this stage. The average true stress difference between the untreated and USRP-treated samples decreased from 122 MPa at the yield strength to the 78 MPa at the peak stress. The difference in true stress was related to the strain hardening exponent n. Khaja et al. [38] attributed the two n values of Inconel 718 samples to the change of deformation mechanism, which transited from planar flow towards cross-slip of dislocations. Sundararaman et al. [39] attributed the drop of the n values to the change in deformation mechanism from dislocation cutting to twinning, caused by the size change of γ″ phases. Some researchers found that the deformation mechanism changes for dislocation movement and the precipitation behavior of δ phase caused the variations in the work hardening exponent [23,33]. In this work, an ultra-fine structure was formed in the near-surface region due to the USRP treatment. During the hot compression process, the microstructure evolution and precipitation behavior of the ultra-fine structure in the near-surface regions may affect the work hardening exponent, thus decreasing the true stress value difference between the untreated and USRP-treated samples.

### 3.2. Microstructure Evolution

**Figure 4:**
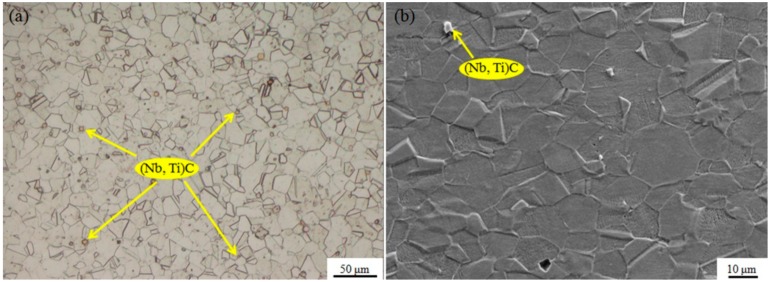
Optical and scanning electron microscope (SEM) morphologies of the as-received Inconel 718 superalloy: (**a**) Optical map, (**b**) SEM map.

**Figure 5:**
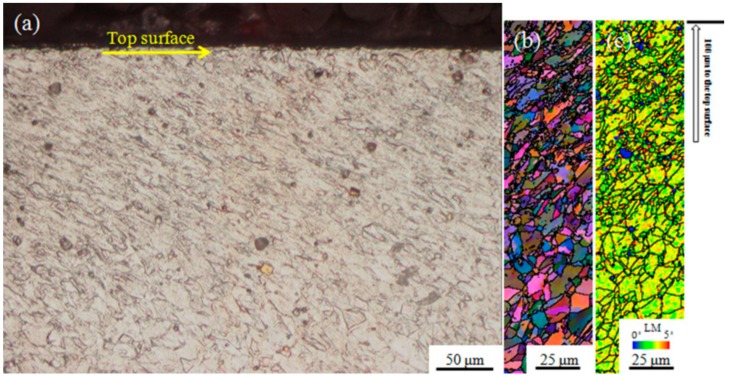
Optical and electron back-scattered diffraction (EBSD) maps of the USRP-treated samples prior to hot compression test: (**a**) Optical map, (**b**) EBSD map of all Euler angles, (**c**) Local mis-orientation (LM) map.

The observed hot compression behaviors were related to microstructural evolution. The characteristics of the original samples were identified for the purpose of monitoring the microstructure evolution after hot compression test. The initial microstructure of the as-received Inconel 718 superalloy is shown in Figure 4. An optical microscopic view (Figure 4a) displayed an equiaxed grain structure with numerous extensive annealing twins. As shown in the SEM micrograph (Figure 4b), there was no significant precipitation (γ″ and δ phase) on the grain boundaries or inside the grain, except for a few uniformly dispersed (Nb, Ti)C particles [7] in the microstructure. To better characterize the microstructure, EBSD was conducted on the USRP-treated samples. The microstructure of the USRP-treated samples prior to hot compression is exhibited in Figure 5. An ultra-fine structure with a thickness of about 200 μm was formed in the near-surface regions due to USRP treatment, as depicted in Figure 5a. During USRP treatment, severe plastic deformation occurred in the near-surface regions due to serious extrusion caused by the ultrasonic strike of the scrollable rolling tip [40]. The dislocations generated by the ultrasonic strikes increased as the number of strikes increased, and accumulated rapidly in the near-surface regions. When the dislocations were accumulated to a certain extent, dislocation walls and dislocation entanglements were formed through dislocation propagation, annihilation, and recombination, which would gradually transform into small-angle subgrain boundaries and deformation twin lamellae. Further straining caused by the ultrasonic strikes created more and more refined subgrains, resulting in randomly distributed ultra-fine grains in the top-surface regions. As for the regions below the top surface, the dislocation density imparted by the ultrasonic strikes was insufficient to reach the critical value to form subgrains. Then, the microstructure of the regions below the top surface was composed of relatively larger grains with high dislocation density, compared with the grains in the top surface regions. As little plastic deformation was delivered to the interior (matrix), the grain size and the dislocation density were not substantially affected. The EBSD map of all Euler angles (Figure 5b) reflects the gradient changes in the microstructure mentioned above. The gradient changes in the dislocation density which can be evaluated by the local mis-orientation (LM) was reflected by the LM map (Figure 5c). A larger LM value represents a higher dislocation density [32]. Due to the good cooling conditions on the self-designed lathe, the temperature rise was not obvious during the USRP treatment. Thus, the temperature after USRP could be considered to be room temperature—the same as before USRP (note that room temperature is much lower than the precipitation temperatures of γ″ and δ phases). Therefore, the USRP-treated samples had the same precipitation state as the as-received samples before hot compression test.

**Figure 6:**
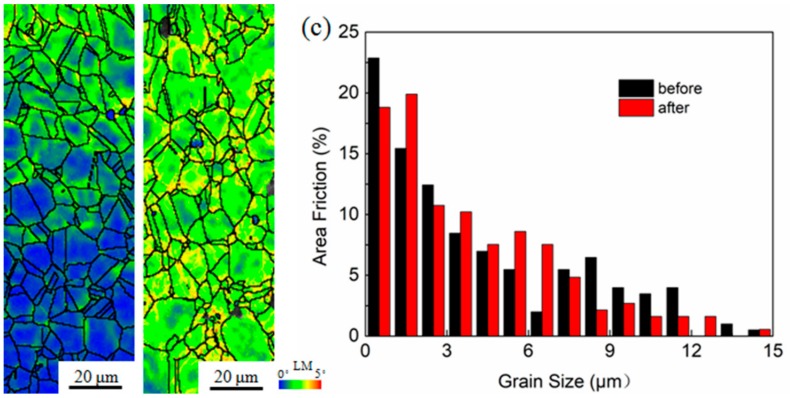
LM maps of the untreated samples (**a**) before and (**b**) after hot compression test, and (**c**) the corresponding grain size distribution map.

After the hot compression test, the microstructural evolution of the untreated and USPR-treated samples was analyzed by EBSD. In this study, only the structural characterization at the true strain rate of 0.001 s^−1^ was given, as the similar deformation behavior of the untreated samples and USRP-treated samples at different true strain rates (0.001, 0.01, 0.1, 1 s^−1^). Figure 6 shows the LM maps of the untreated samples before (Figure 6a) and after (Figure 6b) hot compression test. It can be seen from Figure 6 that the value of LM after compression (Figure 6b) was higher than that before compression (Figure 6a), indicating an enhanced dislocation density. However, after the hot compression test at 700 °C, the untreated sample had the same equiaxed crystal structure and similar grain size distribution (Figure 6c) as before the hot compression test. Therefore, it could be assumed that the input of the axial energy could only increase the dislocation density and was insufficient to break the crystal grains and the twins. In addition, no dynamic recrystallization was found in the untreated samples, leading to the absence of dynamic softening. In the uniform elastic deformation stage shown in Figure 2, most of the input energies were utilized to increase the dislocation density in grains. In the uniform plastic deformation stage, a certain degree of transformation of the grains consumed most of the input energies. As shown in Figure 6b, there was no dynamic recrystallization during the uniform plastic deformation stage. In addition, the dynamic recovery of the Inconel 718 superalloy was very small [41]. The absence of thermal softening (Figure 2) was caused by the above two reasons. This is one of the reasons why the Inconel 718 superalloy can maintain high strength at 700 °C.

**Figure 7:**
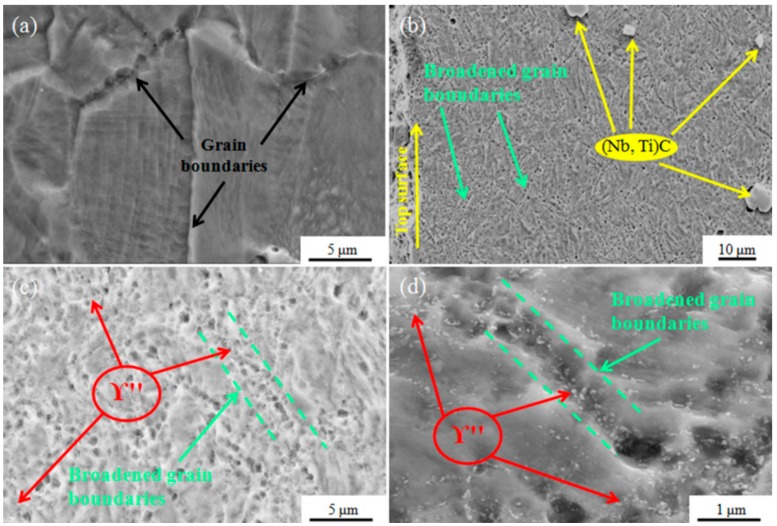
SEM maps of untreated and USRP-treated samples after the hot compression test at the true strain rate of 0.001 s^−1^ and temperature of 700 °C: (**a**) Showing the grain boundaries of the untreated sample, (**b**) Showing the broadened grain boundaries in the near-surface regions of the USRP-treated sample, (**c**,**d**) The precipitation of γ″ phase within broadened grain boundaries in the near-surface regions of the USRP-treated sample.

The SEM maps of untreated samples and USRP-treated samples after hot compression tests at the true strain rate of 0.001s^−1^ and temperature of 700 °C are shown in Figure 7. No precipitate was observed in the grain boundaries of the untreated sample after the hot compression test, as shown in Figure 7a. Many broadened grain boundaries existed on the near-surface regions of the USRP-treated samples, as depicted in Figure 7b. The magnified morphologies in Figure 7c,d demonstrate the broadened characteristic with a width of about 1 μm of the broadened grain boundaries. In addition, many γ″ phases with size less than 100 nm were precipitated on the broadened grain boundaries (Figure 7c,d). Generally, the nucleation process of the γ″ phase was significantly affected by nucleation positions. Defects such as grain boundaries, twins, and dislocations could act as favorable sites for γ″-phase nucleation. As stated above, many broadened grain boundaries, dislocation entanglements, and dislocation walls were formed in the near-surface regions due to the USRP treatment. These defects can provide numerous nucleation sites for γ″ phase. During the hot compression test, the nucleation process involves a large amount of free energy changes that can be simplified as [3]:

(3) $$\Delta G=-\Delta G_{V}+\Delta G_{\gamma }+\Delta G_{S},$$

where ΔG represents the global free energy changes during the nucleation process, $-\Delta G_{V}$ represents the decrease of volume free energy, $\Delta G_{\gamma }$ reflects the increase of interfacial free energy, and $\Delta G_{S}$ reflects the increase of misfit strain energy [3]. For the near-surface regions of the USRP-treated sample, γ″ phase would preferentially nucleate on the broadened grain boundaries, as the broadened grain boundaries could reduce $\Delta G_{\gamma }$ and the piled-up dislocations within broadened grain boundaries could lower the $\Delta G_{S}$. Based on Equation (3), the nucleation on dislocations within broadened grain boundaries could release more energy, resulting in the decrease of the activation energy barrier. In addition, the nucleation period of γ″ phase in the Inconel 718 superalloy was considerably short, as reported by Fisk et al. [4]. Therefore, numerous precipitates were nucleated within the broadened grain boundaries in a few minutes during the hot compression test (Figure 7c,d). Similar precipitation behaviors were also reported in other references [5,7,8]. The precipitation of the γ″ phase could enhance the strength of Inconel 718 superalloy at high temperature [42]. As for the untreated samples and the matrix regions of the USRP-treated samples, the dislocation density that accumulated during the uniform elastic deformation was far from the extent required to break the grain boundaries. It was insufficient to break the activation energy barrier for the nucleation of γ″ phase at any position. As a result, no γ″ phase was observed on the grain boundaries (Figure 7a).

**Figure 8:**
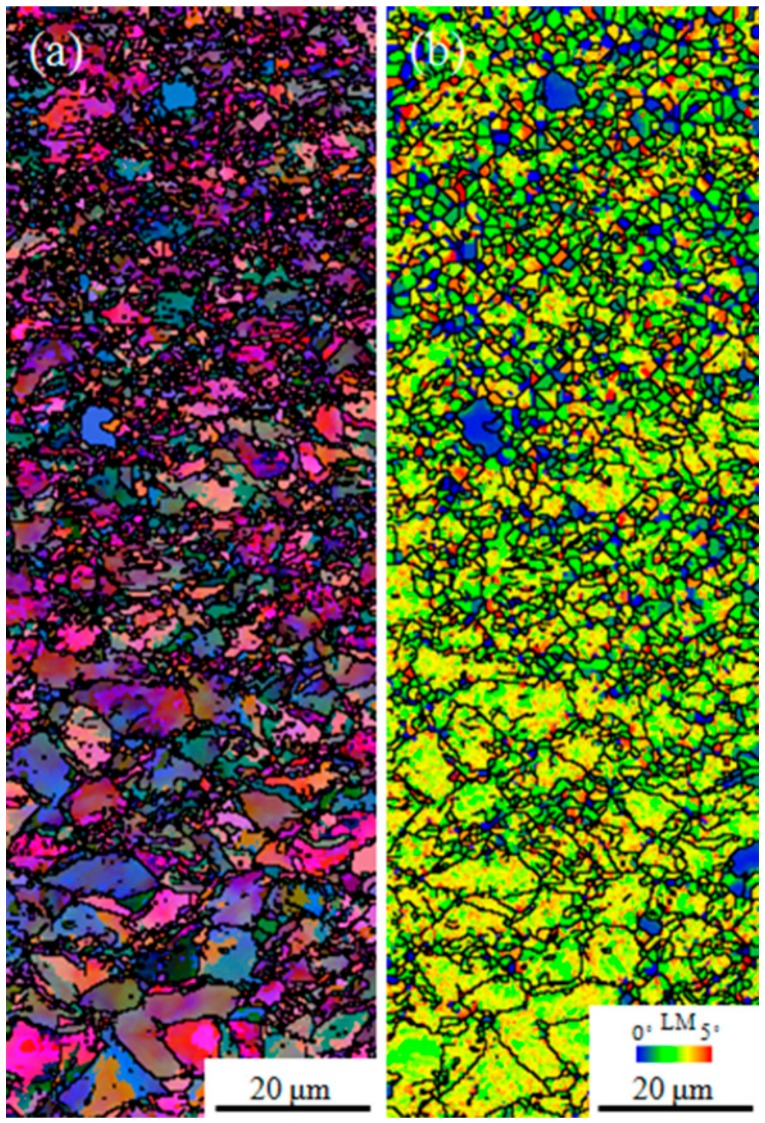
EBSD maps of the USRP-treated sample after hot compression test at the true strain rate of 0.001 s^−1^ and temperature of 700 °C: (**a**) all Euler angles map, (**b**) LM map.

The EBSD maps of the USRP-treated sample after the hot compression test at the true strain rate of 0.001 s^−1^ are shown in Figure 8. Compared with USRP-treated samples before the hot compression test (Figure 5b,c), the number of ultra-fine recrystallized grains in the near-surface regions of Figure 8 was significantly enhanced. The average grain size in the near-surface regions of the USRP-treated samples before (Figure 5b,c) and after (Figure 8) the hot compression test at the true strain rate of 0.001 s^−1^ was calculated to be 1.69 and 0.75 μm, respectively. In the uniform elastic deformation stage, the dislocation density was increased in the matrix grains due to the energy input. In the top-surface regions, the presence of ultra-fine grains, high-density dislocation walls, and dislocation entanglements caused by USRP treatment greatly hindered the further increase of the dislocation density, which means that more energy input/high strength was required. In the regions below the top surface, the dislocation density was much higher than that in the matrix, which means more energy/strength input to increase the dislocation density than the matrix, even though the dislocation density was insufficient to reach the critical value to form subgrains. That is why the true stress of the USRP-treated samples was higher than that of the untreated samples during the uniform elastic deformation stage (Figure 3).

In the uniform plastic deformation stage, the dislocation density in the matrix remained in an increased state during the hot compression process. In the top-surface regions, the increase of dislocation density facilitated the transition from the dislocation walls and dislocation entanglements to ultra-fine grains through dislocation propagation, slip, annihilation, and recombination. In the regions below the top surface, the dislocation density was sufficient to reach the critical value to form subgrains due to the increase of dislocation density. Therefore, many ultra-fine recrystallized grains were formed in the regions below the top surface, as shown in Figure 8a. Figure 8b exhibits the LM map of a USRP-treated sample after the hot compression test. Compared with Figure 5c, Figure 8b contains more complete grains with low LM values (shown as blue color), which is in agreement with the promotion of recrystallization. The new ultra-fine recrystallized grains were formed along the initial grain boundaries, instead of inside the original grains. A similar phenomenon was observed by Mostafa [41] and Wang et al. [43]. As a result, the thermal softening of the USRP-treated sample was enhanced due to the increase of ultra-fine recrystallized grains in the top-surface regions and the generation of ultra-fine recrystallized grains in the regions below the top surface during the uniform plastic deformation stage. Therefore, the difference between the true stress values of the USRP-treated samples and untreated samples at the uniform plastic deformation stage decreased with the increase of the true strain.

## 4. Conclusions

The effect of USRP on the hot compression behavior of Inconel 718 superalloy was investigated by adopting a series of hot compression tests at 700 °C. The ability of Inconel 718 superalloy to withstand axial compression at 700 °C was enhanced by USRP treatment. After axial hot compression at 700 °C, compared with untreated samples, the size of the matrix grains was slightly decreased and the volume fraction of ultra-fine recrystallized grains in the near-surface regions was increased. In addition, a large number of nano-sized γ″ phases precipitated within the broadened grain boundaries in the near-surface regions. The ultra-fine microstructure in the near-surface regions induced by USRP, combined with the precipitation of nano-γ″ phases within broadened grain boundaries and the increased volume fraction of ultra-fine recrystallized grains in the near-surface regions, were responsible for the enhanced ability to withstand axial compression at 700 °C.

## Figures and Tables

**Table 1:** Ultrasonic surface rolling process (USRP) processing parameters.

Condition	Rolling Line Speed	Feeding Rate	Frequency	Static Force
USRP	12 m/min	13 mm/min	27 kHz	934 N

**Table 2:** The peak stress (MPa) of the true stress–strain curves.

	Type	0.001	0.01	0.1	1
Strain Rate (s^−1^)					
Untreated sample		929.8	998.4	985.0	952
USRP-treated sample		1015.1	1054.4	1067.5	1039.5
